# Development and Validation of Machine Learning–Based Models to Predict In-Hospital Mortality in Life-Threatening Ventricular Arrhythmias: Retrospective Cohort Study

**DOI:** 10.2196/47664

**Published:** 2023-11-15

**Authors:** Le Li, Ligang Ding, Zhuxin Zhang, Likun Zhou, Zhenhao Zhang, Yulong Xiong, Zhao Hu, Yan Yao

**Affiliations:** 1 National Center for Cardiovascular Diseases, Fu Wai Hospital Peking Union Medical College Chinese Academy of Medical Sciences Beijing China

**Keywords:** life-threatening ventricular arrhythmia, mortality, prediction model, machine learning, critical care, cardiac, mortality

## Abstract

**Background:**

Life-threatening ventricular arrhythmias (LTVAs) are main causes of sudden cardiac arrest and are highly associated with an increased risk of mortality. A prediction model that enables early identification of the high-risk individuals is still lacking.

**Objective:**

We aimed to build machine learning (ML)–based models to predict in-hospital mortality in patients with LTVA.

**Methods:**

A total of 3140 patients with LTVA were randomly divided into training (n=2512, 80%) and internal validation (n=628, 20%) sets. Moreover, data of 2851 patients from another database were collected as the external validation set. The primary output was the probability of in-hospital mortality. The discriminatory ability was evaluated by the area under the receiver operating characteristic curve (AUC). The prediction performances of 5 ML algorithms were compared with 2 conventional scoring systems, namely, the simplified acute physiology score (SAPS-II) and the logistic organ dysfunction system (LODS).

**Results:**

The prediction performance of the 5 ML algorithms significantly outperformed the traditional models in predicting in-hospital mortality. CatBoost showed the highest AUC of 90.5% (95% CI 87.5%-93.5%), followed by LightGBM with an AUC of 90.1% (95% CI 86.8%-93.4%). Conversely, the predictive values of SAPS-II and LODS were unsatisfactory, with AUCs of 78.0% (95% CI 71.7%-84.3%) and 74.9% (95% CI 67.2%-82.6%), respectively. The superiority of ML-based models was also shown in the external validation set.

**Conclusions:**

ML-based models could improve the predictive values of in-hospital mortality prediction for patients with LTVA compared with traditional scoring systems.

## Introduction

Sudden cardiac arrest (SCA) is associated with unacceptable high mortality rates worldwide [1-3]. It is estimated that 275,000 people in Europe present with SCA treated by emergency medical services (EMSs) each year, only 29,000 of whom survive to hospital discharge [4]. Moreover, the EMS-treated SCA incidences in the United States, Canada, and China were about 64.4, 54.7, and 71.2 per 100,000 person years, respectively [5,6]. Life-threatening ventricular arrhythmias (LTVAs), which occur with severely depressed ventricular function and an unstable hemodynamics state, are the main causes of SCA [7]. The high morbidity and mortality of LTVA cause a substantial economic burden and a serious health burden for EMSs [8,9]. Risk stratification and mortality assessment for patients with LTVA provide not only essential strategies for clinical decision-making but also practical information for health policy and insurance services [10,11].

Because patients with LTVA have a poor prognosis, early recognition of high-risk individuals is critical for timely interventions as well as intensive care and monitoring. The simplified acute physiology score (SAPS-II) [12] and the logistic organ dysfunction system (LODS) [13] are traditional severity assessment systems that can predict the risk of death in general patients who received EMS. The LODS and SAPS-II models are comprehensive scoring systems used to evaluate the general conditions of patients and are limited by weak specificity. Moreover, several specific risk scores have been developed to perform SCA prediction in patients with hypertrophic cardiomyopathy [14] and arrhythmogenic cardiomyopathy [15]. However, a prediction model assessing the prognosis in patients with LTVA is still lacking.

Recently, machine learning (ML) algorithms have been used to build prediction models in clinical medicine and have been demonstrated to have more favorable predictive performance than traditional models [16-18]. In this study, we aimed to develop and validate ML-based models to predict in-hospital mortality in critically ill patients with LTVA. Additionally, the comparisons of the ML models and the traditional severity assessment systems SAPS-II and LODS were performed to further demonstrate the predictive value of ML algorithms.

## Methods

### Source of Data

Patients’ data collected from the Medical Information Mart for Intensive Care IV (MIMIC-IV, version 2.0) database were used to perform model training. The MIMIC-IV is a large critical care database that contains data from more than 200,000 individuals who were admitted to various intensive care units (ICUs) at the Beth Israel Deaconess Medical Center between 2008 and 2019 [19]. In addition, data from the eICU Collaborative Research Database (eICU, version 2.0), which is a multicenter critical care database with high granularity data for over 200,000 admissions to ICUs [20], were used to conduct the external validation.

### Patient and Data Collection

Patients with LTVA in the hospital were included in this study. LTVAs were defined as documented sustained ventricular tachycardia (duration >30 seconds) or ventricular fibrillation. Patients aged <18 years or without adequate data, including demographic variables (eg, age, sex) and hospitalization details (eg, time of stay in the ICU, in-hospital mortality), were excluded. The data were collected from the database using Structure Query Language (PostgreSQL version 13.0). The variables included in this study could be divided into several categories: (1) demographics, including age, sex, race, height, and weight; (2) vital signs, including temperature, heart rate, blood pressure, respiratory rate, Glasgow coma scale (GCS), and urine output; (3) blood gas test, including pH, partial pressure of oxygen, partial pressure of carbon dioxide, and other parameters; (4) laboratory results, including white blood cell count, red blood cell count (RBC), creatinine, blood urea nitrogen, and other parameters; (5) comorbidities, including hypertension, diabetes, acute heart failure, myocardial infarction, and others; and (6) important interventions, including mechanical ventilation, renal replacement therapy, inotropic agents, and antibiotics. For variables with several records (eg, vital signs and laboratory tests), the minimum and maximum values within the first 24 hours after ICU admission were calculated, which may reflect the truly general conditions of the patients admitted to the ICU.

Several methods were introduced to handle the issues of extreme and missing data. Variables with >40% missing values were excluded from this study. Mean imputation was performed to fill in missing information for variables with <5% missing data. The multiple imputation was performed using the R multivariate imputation by chained equation (MICE) package for the variables with 5%-40% missing data [21].

### Outcome Variables and Predictors

The primary outcome of interest was all-cause in-hospital mortality. The predictor variables considered in this study were selected based on a feature selection process (Multimedia Appendix 1).

### Model Development and Validation

In this study, the ML-based prediction models were established using the following steps:

1. Feature selection: we applied CatBoost and LightGBM to initially evaluate the importance of individual features. Moreover, least absolute shrinkage and selection operator (LASSO) analysis was used to select the key predictors. LASSO is a form of L1 regularization that adds the absolute magnitude of feature coefficients as the penalty term rather than the squared magnitude that is typically used [22]. After LASSO analysis, features with a zero coefficient were considered redundant and eliminated from the model fitting. As a result, automatic variable selection while simultaneously fitting the model could be realized.
2. Algorithm selection and optimization: a total of 5 commonly used ML algorithms, including CatBoost, LightGBM, back propagation–neural network, random forest, and logistic regression, were applied to build the prediction models. We used the grid search strategy to identify the optimal combination of hyperparameters of these models to improve the prediction performance. Ten-fold stratified cross-validation was performed inside each grid option, and the optimal hyperparameter set was chosen based on the model in the grid search with the highest F1 score.
3. Model fitting: before model fitting, a combination of the synthetic minority oversampling technique and undersampling was used to overcome the class-imbalance issue in this study. Qualified data from the MIMIC-IV database were randomly split into a training set (80% of the sample) and an internal validation set (20% of the sample) using the *“train_test_split”* function provided by Scikit-learn. As a result, the ML-based prediction models were developed.

In this study, the prediction models were validated both internally and externally. The discriminatory ability of the prediction model was evaluated by using the area under the receiver operating characteristic curve (AUC). The calibrated ability was qualitatively and quantitively assessed by the calibration curve and the Brier score, respectively. Calibration reflects the extent to which the predicted probabilities and actual probabilities agree and is qualitatively and quantitatively evaluated through the calibration curve and Brier score, respectively. The Brier score is calculated based on the Euclidean distance between the actual outcome and the predicted probability assigned to the outcome for each observation, with low values being desirable. In addition, decision curve analysis was used to demonstrate the decision benefit based on the models.

Several commonly used indices, including the AUC, sensitivity, specificity, and F1 score, were introduced to quantitively evaluate the predictive values of the models. To further demonstrate the favorable prediction performances of the ML-based models, the ML models and traditional severity assessment systems SAPS-II and LODS were compared in this study.

### Model Interpretation

The black box characteristic of ML models makes it difficult to interpret how an ML algorithm could perform accurate prediction in clinical medicine settings. Accordingly, the Shapley additive explanations (SHAP) value was introduced in this study. SHAP was used to interpret the results from a predictive model. The interpretation was based on the SHAP value for each feature, representing the contribution of a feature to the predicted risk of the event. A positive SHAP value indicated that the corresponding feature contributed to a higher risk of the event, whereas a negative SHAP value indicated that the corresponding feature led to a lower risk of the event. The magnitude of SHAP values represented the contribution of that feature toward prediction performance [23]. The SHAP summary plot was constructed to interpret and rank the significance of input features based on the mean absolute SHAP values of each feature. The SHAP dependency plot was used to understand how a single feature could affect the output of the prediction models.

### Statistical Analysis

The Kolmogorov-Smirnov test was used to evaluate the normal distribution of the data. Continuous variables were expressed as the mean (SD) or median (IQR) depending on the distribution of the data. Categorical data were summarized as frequencies and percentages. Baseline clinical characteristics were compared between the survival and nonsurvival groups using a *t* test or Welch *t* test for continuous variables and the chi-square test for dichotomous variables. The prediction performances of the different models were compared using a Delong test. All statistical tests were 2-sided, and *P*<.05 was considered statistically significant. Statistical analyses were performed in R (version 4.0.4, R Foundation for Statistical Computing). Python (version 3.9.0, Python Software Foundation) was used to conduct the ML-relevant processes.

### Ethics Approval

This study was an analysis of a third-party, anonymized, publicly available database with pre-existing institutional review board approval, and informed consent from our institution was exempted. Data usage was approved by the review board of PhysioNet (authorization code: 35965741). The study was reported according to the recommendations of the TRIPOD (Transparent Reporting of a Multivariable Prediction Model for Individual Prognosis or Diagnosis) statement [24].

## Results

### Baseline Characteristics

**Figure 1:**
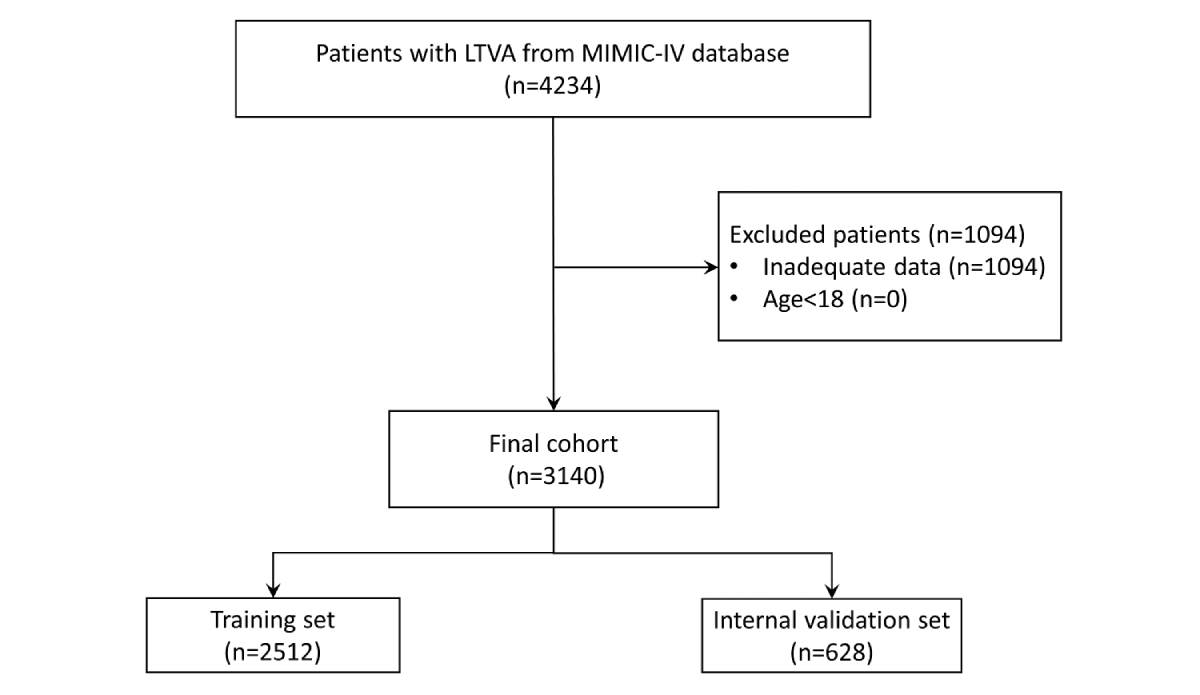
Flowchart. LTVA: life-threatening ventricular arrhythmias; MIMIC-IV: Medical Information Mart for Intensive Care IV.

We included 3140 critically ill patients with LTVA from the MIMIC-IV database, of whom 632 (20.1%) individuals died during hospitalization (Figure 1). Compared with the survival group, nonsurvival patients were older (nonsurvival: mean 71.0, SD 14.2 years; survival: mean 67.9, SD 14.9 years; *P*<.001) and had lower GCS scores (nonsurvival: mean 10.4, SD 4.8; survival: mean 13.0, SD 3.2: *P*<.001). Moreover, nonsurvival patients were prone to having unstable vital signs. Furthermore, the data of 2851 patients from another database were used to perform the external validation. The baseline characteristics of the 2 cohorts are shown in Multimedia Appendix 1.

### Model Development and Validation

**Figure 2:**
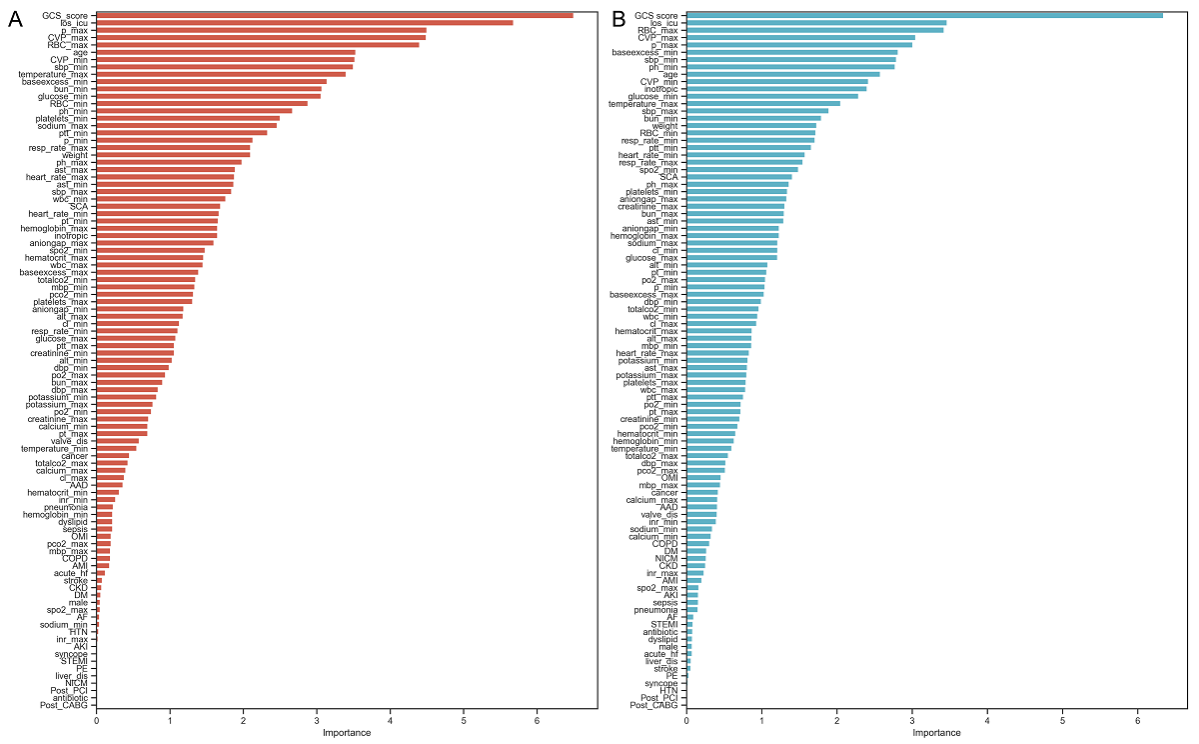
Feature importance evaluated using the (A) CatBoost model or (B) LightGBM model.

A total of 94 features were initially selected as the potential predictors (Multimedia Appendix 2). Feature importance, evaluated using the CatBoost and LightGBM models, is shown in Figure 2. We found that the GCS score, maximum RBC (RBC_max), and length of stay (LOS) in hospital prior to LTVA were the most predictive features in both the CatBoost and LightGBM models, indicating the certain stability. In the LASSO analysis, the coefficients of 54 variables were shrunk to zero (Multimedia Appendix 3). Consequently, we retained 40 key features for the remaining analyses (Multimedia Appendix 4).

**Figure 3:**
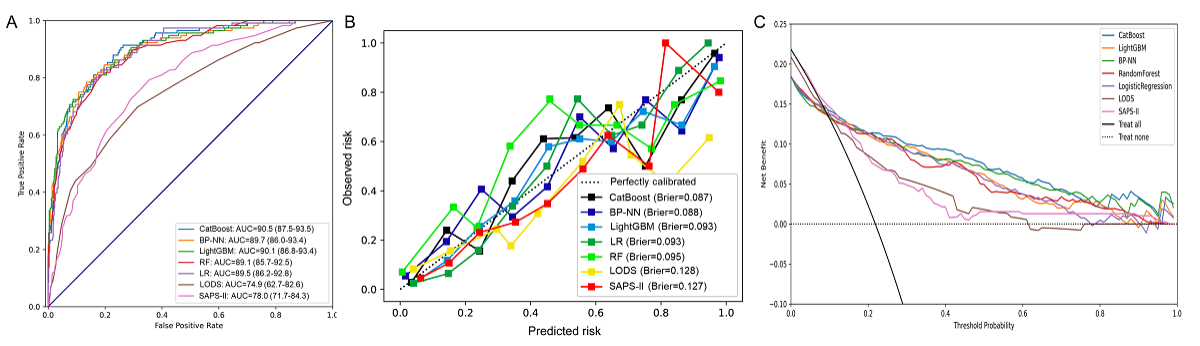
Model evaluation. (A) The receiver operating characteristic curves of the different models. (B) Calibration curves and Brier scores of the different models. The x-axis is the predicted probability of outcomes, and the y-axis is the true probability of outcomes. In the case of perfect calibration, all groups of predicted probabilities fit close to the dotted diagonal line corresponding to an intercept of 0 and a slope of 1 for the calibration plot. (C) Decision curve analysis of the different models. The x-axis is the threshold probability, which refers to the point at which a patient considers the benefit of treatment for an intermediate- to high-risk event equivalent to the harm of overtreatment for a low-risk event and thus reflects how the patient weighs the benefits and harms associated with this decision. The y-axis is the net benefit, which is defined as the minimum probability of event at which further intervention would be warranted (net benefit = true positive rate – [false positive rate × weighting factor]). AUC: area under the receiver operating characteristic curve; BP-NN: back propagated-neural network; LODS: logistic organ dysfunction system; LR: logistic regression; RF: random forest; SAPS-II: simplified acute physiology score.

As we mentioned above, 5 widely used ML algorithms were used to build the prediction models with the grid search strategy to optimize the ML-based models. Multimedia Appendix 5 summarizes the characteristics of the 5 ML algorithms. The ML-based models showed favorable discriminatory ability, with AUCs of 90.5% (95% CI 87.5%-93.5%), 89.7% (95% CI 86.0%-93.4%), 90.1 (95% CI 86.8%-93.4%), 89.1% (95% CI 85.7%-92.5%), and 89.5% (95% CI 86.2%-92.8%) for the CatBoost, back propagation–neural network, LightGBM, random forest, and logistic regression models, respectively. Conversely, the traditional models presented with ordinary prediction performance, with AUCs of 74.9% (95% CI 67.2%-82.6%) and 78.0% (95% CI 71.7%-84.3%) for LODS and SAPS-II, respectively (Figure 3A). The detailed prediction results for each model are shown in Table 1. We found that the AUCs of the 5 ML algorithms were all significantly higher than those of the SAPS-II and LODS models (all *P*<.001; Multimedia Appendix 6).

**Table 1:** Prediction performances of the different models.

Model		AUC^a^, % (95% CI)	SENS^b^, % (95% CI)	SPEC^c^, % (95% CI)	F1 score, % (95% CI)
**Internal validation**					
	CatBoost	90.5 (87.5-93.5)	73.3 (65.4-81.3)	93.8 (92.2-95.4)	77.5 (70.5-84.5)
	LightGBM	90.1 (86.8-93.4)	72.4 (68.1-76.7)	94.3 (92.4-96.2)	76.4 (69.2-83.6)
	RF^d^	89.1 (85.7-92.5)	64.8 (56.7-73.0)	97.6 (96.0-99.2)	75.7 (68.5-82.9)
	LR^e^	89.5 (86.2-92.8)	67.6 (60.0-75.3)	95.1 (93.5-96.8)	75.1 (66.5-82.0)
	BP-NN^f^	89.7 (86.0-93.4)	69.3 (61.9-76.7)	96.5 (94.5-98.5)	77.7 (71.2-84.1)
	LODS^g^	74.9 (72.3-80.5)	55.5 (45.5-65.5)	96.3 (93.6-99.0)	68.3 (58.9-77.7)
	SAPS-II^h^	78.0 (71.4-79.0)	58.8 (50.3-67.1)	93.9 (91.7-96.2)	69.0 (60.0-78.1)
**External validation**					
	CatBoost	87.2 (83.6-90.8)	60.7 (54.9-66.5)	96.6 (95.0-98.2)	71.8 (67.7-75.9)
	LightGBM	84.9 (81.9-87.9)	67.1 (60.6-73.6)	94.2 (92.4-96.0)	73.9 (70.1-77.7)
	RF	86.0 (83.4-88.6)	58.2 (52.5-63.9)	96.8 (95.6-98.0)	70.1 (65.8-74.4)
	LR	81.1 (77.5-84.7)	57.2 (51.5-62.9)	94.3 (93.1-95.5)	76.4 (69.2-83.6)
	BP-NN	85.6 (82.8-88.4)	64.3 (57.8-70.8)	94.8 (93.0-96.6)	65.8 (61.4-70.1)
	LODS	73.7 (68.8-77.6)	51.0 (36.1-65.9)	96.6 (94.4-98.8)	64.8 (57.4-72.2)
	SAPS-II	71.9 (63.8-80.0)	46.3 (37.2-55.4)	92.3 (88.9-95.7)	55.8 (46.2-65.4)

^a^AUC: area under the receiver operating characteristic curve.

^b^SENS: sensitivity.

^c^SPEC: specificity.

^d^RF: random forest.

^e^LR: logistic regression.

^f^BP-NN: back propagated–neural network.

^g^LODS: logistic organ dysfunction system.

^h^SAPS-II: simplified acute physiology score.

The 5 ML-based models showed excellent fit, with all Brier scores <0.1. However, the SAPS-II and LODS models largely deviated from the true events (Figure 3B). In the decision curve analysis, the net benefits of all ML-based models outperformed the traditional models when the threshold probability was between 0.2 and 0.7, and the CatBoost model showed the best decision benefit compared with the others (Figure 3C).

In the external validation set, prediction performances of the 5 ML-based models were also significantly superior to the SAPS-II and LODS models. The AUCs of the SAPS-II and LODS models were only 73.7% (95% CI 68.8%-77.6%) and 71.9% (95% CI 63.8%-80.0%), respectively (Multimedia Appendix 7 and Table 1). Accordingly, we suggest that the performance of the ML models is significantly superior to that of the traditional prognosis assessment systems for critically ill patients with LTVA.

### Model Interpretation

**Figure 4:**
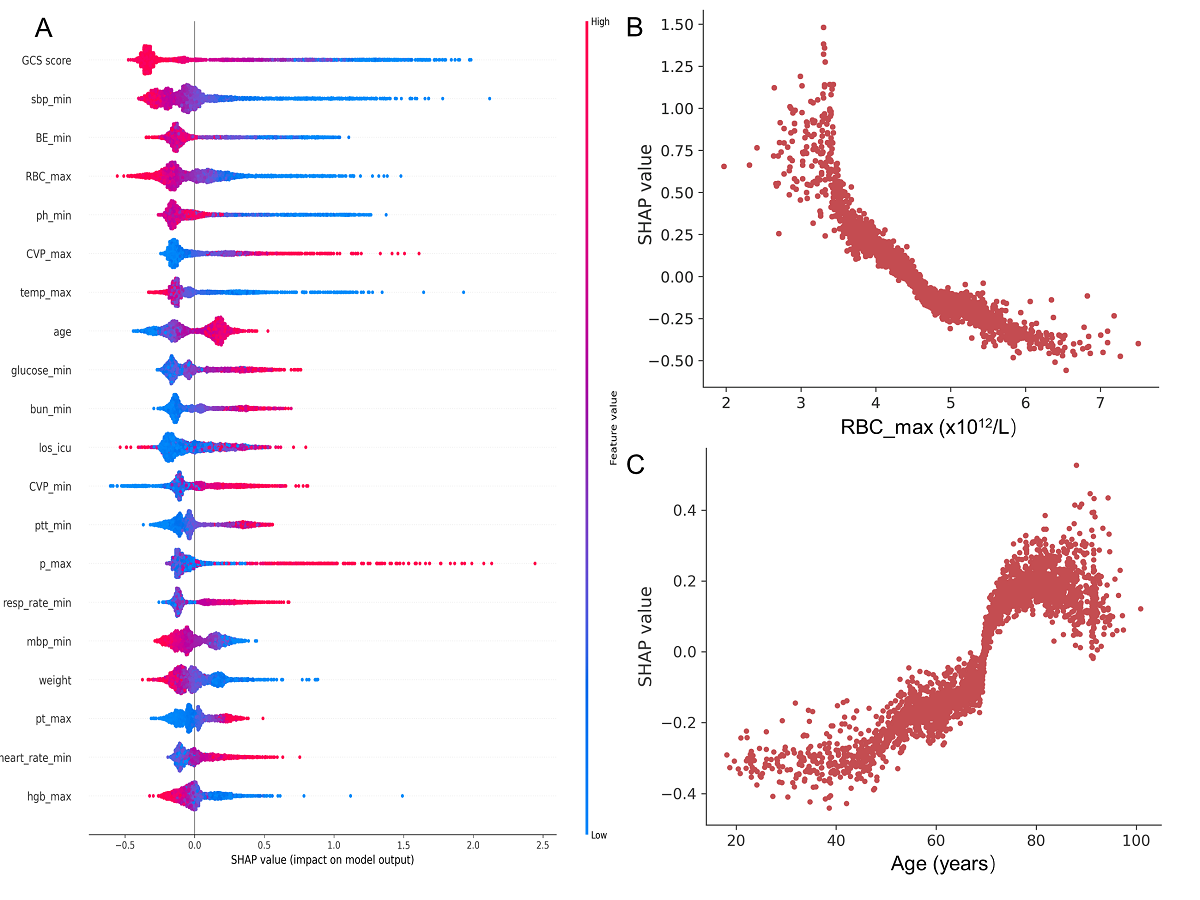
Model interpretation based on the SHAP value. (A) SHAP summary plot. A dot is created for each feature attribution value for the model of each patient, and thus one patient is allocated one dot on the line for each feature. Dots are colored according to the values of features for the respective patient and accumulate vertically to depict density. The blue to red color of the y-axis represents the feature value (red: high; blue: low). The x-axis measures the impacts on the model output (right: positive; left: negative); the higher the SHAP value of a feature, the higher the probability of in-hospital mortality development. (B, C) SHAP dependency plots. SHAP values for specific features exceed zero, representing an increased risk of in-hospital mortality. RBC_max: the maximum red blood cell count; SHAP: Shapley additive explanation.

To interpret the ML-based models, the SHAP method was used in this study. Specifically, we calculated the absolute mean SHAP values for each variable based on the CatBoost model applied to the internal validation set. For instance, with decreasing GCS scores and RBC_max values, the risk of in-hospital mortality of patients with LTVA increased. Moreover, the probability of in-hospital mortality increased with age and maximum central venous pressure values (Figure 4A). Additionally, the SHAP dependency plot showed how different values of each feature affect the SHAP value and influence the output of the prediction models (Figure 4B and C). SHAP values for specific features exceeding zero represent an increased risk of in-hospital mortality.

## Discussion

The primary finding of this study demonstrates that ML algorithms significantly enhance the prognosis prediction performance for patients with LTVA. Compared with traditional scoring systems, including SAPS-II and LODS, the ML-based models enabled better prediction of in-hospital mortality in critically ill patients with LTVA.

LTVAs are the main cause of SCA and are highly related to an increased risk of in-hospital mortality. Several prediction models have been developed to predict SCA. O’Mahony et al [25] established a model that provided accurate individualized estimates for the probability of SCA in patients with hypertrophic myocardiopathy using 8 commonly used clinical parameters, with a C-statistic of 0.70. Adabag and Langsetmo [26] built a SCA risk prediction model that could accurately predict SCA events in heart failure with preserved ejection fraction, with a C-statistic of 0.74. However, a model to predict outcomes in patients with LTVA is still lacking. The application value of ML algorithms in improving the mortality prediction performance for patients with LTVA remains unclear. Thus, we developed and validated ML-based models to precisely predict in-hospital mortality in patients with LTVA.

There are several severity assessment systems that could be used to predict outcomes of patients who are critically ill. The application values of SAPS-II and LODS in predicting the prognosis in patients who received EMS have been demonstrated in previous studies [27-29]. ML-based models could further improve the prediction performance to predict the mortality in certain subsets [30,31]. The ML algorithms also exhibited superiority over the SAPS-II and LODS models in this study. SAPS-II incorporates 12 physiological variables, age, type of admission, and 3 underlying disease variables, while LODS identifies levels of organ dysfunction for 6 organ systems (ie, neurologic, cardiovascular, renal, pulmonary, hematologic, and hepatic). In contrast, our developed models included additional important features, such as GCS score, RBC, and central venous pressure, which may have contributed to our improved prediction performance. Therefore, the enhanced performance of the ML models in our study may be attributed to their ability to effectively capture intricate relationships and patterns within a more extensive set of variables.

Of the 40 selected variables, we found that GCS score, RBC_max, and LOS in hospital were the most important predictors. The GCS score includes assessments of motor, verbal, and eye responses, which could reflect the level of consciousness. A previous study reported that the GCS score could be used to predict mortality in patients who are critically ill [32], which is consistent with our findings. Anemia on admission is also associated with an increased mortality in critical care patients [33]. We also found there was an inverse relationship between RBC and in-hospital mortality. Moreover, LOS was a key variable for predicting mortality, as expected. Other predictors included vital signs, hepatic and renal function tests, and relevant comorbidities. Although abnormal potassium levels and the use of macrolide antibiotics are known risk factors for LTVA and may increase the risk of mortality [34,35], we observed weak contributions of these features to our predictive models. This could be attributed to the complex interplay between multiple factors in the development of arrhythmias and the limitations imposed by the sample size of our study.

In addition, the ML-based models in this study had certain interpretability. Many of the features used to perform mortality risk prediction in this study were tangible, and some of them had been proven intimately correlated with mortality in patients who are critically ill. The SHAP method, which could provide a visual interpretation of the ML-based models at the global and local levels, was introduced in this study. Specifically, the SHAP summary plot, which interpreted and ranked the significance of input features, and the SHAP dependency plot, which explained how a single feature affected the output of the ML-based models, were presented in this study.

The prediction models in this study enable early and accurate selection of patients at high risk of in-hospital death, which may be conducive to risk stratification, clinical decision-making, and the improvement of prognosis in patients with LTVA. On the other hand, our prediction models were developed based on critical care cohorts. Essentially, sicker patients with more multisystem derangements are anticipated to have poorer prognosis. Considering their satisfactory specificities, our prediction models may help to identify subgroups of patients with LTVA treated with EMSs who are not anticipated to have an adverse prognosis. It is useful to avoid wasting medical services, such as through unnecessary intensive monitoring and aggressive therapies in the ICU.

Although we demonstrated the predictive value of ML algorithms in predicting mortality in critical patients with LTVA, some limitations should be acknowledged. First, all data involved in this study were retrospectively collected from public databases, and further prospective studies are needed to confirm the findings. Second, some important variables, such as cardiac troponin and ejection fraction, were excluded from this study due to unacceptably high rates of missing values, which may be a potential source of bias. Third, the prediction models were developed based on critical care cohorts, which may affect application to the general population.

In summary, the presented ML-based models exhibited better predictive values than did traditional severity assessment systems, such as SAPS-II and LODS, in predicting the mortality of critical patients with LTVA. Our findings indicate that ML algorithms could be used to improve model performance for predicting outcomes of patients with LTVA. Future prospective studies are needed to confirm the findings.

## Appendix

Multimedia Appendix 1 Baseline characteristics.

Multimedia Appendix 2 All variables used in the machine learning models.

Multimedia Appendix 3 Variables excluded after feature selection by least absolute shrinkage and selection operator (LASSO).

Multimedia Appendix 4 Feature selection by least absolute shrinkage and selection operator (LASSO) analysis. (A) Plots for LASSO analysis coefficients. (B) Cross-validation plot for the penalty term.

Multimedia Appendix 5 Characteristics of the included machine learning algorithms.

Multimedia Appendix 6 Comparisons of the area under the receiver operating characteristic curve of different models using the Delong test.

Multimedia Appendix 7 External validation of different models.

## Abbreviations

AUC: area under the receiver operating characteristic curve
EMS: emergency medical services
GCS: Glasgow coma scale
ICU: intensive care unit
LASSO: least absolute shrinkage and selection operator
LODS: logistic organ dysfunction system
LOS: length of stay
LTVA: life-threatening ventricular arrhythmia
MICE: multivariate imputation by chained equation
MIMIC-IV: Medical Information Mart for Intensive Care IV
ML: machine learning
RBC: red blood cell count
RBC_max: maximum red blood cell count
SAPS-II: simplified acute physiology score
SCA: sudden cardiac arrest
SHAP: Shapley additive explanations
TRIPOD: Transparent Reporting of a Multivariable Prediction Model for Individual Prognosis or Diagnosis

## Data Availability

The data sets used and analyzed during this study are available from the corresponding author upon reasonable request.
