# Supplementary material for: Development and Validation of Machine Learning–Based Models to Predict In-Hospital Mortality in Life-Threatening Ventricular Arrhythmias: Retrospective Cohort Study
Source: J Med Internet Res. 2023 Nov 15;25:e47664. doi: 10.2196/47664 (PMC10687678; doi:10.2196/47664)
Supplement: Multimedia Appendix 1 [file jmir_v25i1e47664_app1.docx]

| Multimedia Appendix 1. Baseline Characteristic. | | | | | | |
| --- | --- | --- | --- | --- | --- | --- |
|  | Training cohort (n = 3140) | | | Validation cohort (n = 2851) | | |
| Variables | Survival  (n = 2508) | Non-Survival  (n = 632) | *P* value | Survival  (n = 2399) | Non-Survival  (n = 525) | *P* value |
| Age, year | 69 (59–79) | 72 (62–81) | < 0.001 | 66 (55–75) | 70 (60–78) | < 0.001 |
| Weight, Kg | 84.7 ± 21.2 | 81.5 ± 21.7 | < 0.001 | 88.2 ± 25.5 | 85.7 ± 26.2 | 0.046 |
| GCS score | 14 (12–15) | 13 (6–15) | < 0.001 | 15 (14–15) | 11 (3–15) | < 0.001 |
| LOS_ICU | 4.00 ± 5.10 | 5.44 ± 6.48 | < 0.001 | 5.57 ± 6.37 | 6.29 ± 7.15 | 0.024 |
| Acute HF, % | 349 (13.9%) | 74 (11.7%) | 0.149 | 439 (18.3%) | 95 (18.1%) | 0.913 |
| OMI, % | 539 (21.5%) | 200 (31.6%) | < 0.001 | 496 (20.7%) | 97 (18.5%) | 0.256 |
| SCA, % | 188 (7.5%) | 70 (11.1%) | < 0.001 | 311 (13.0%) | 237 (45.1%) | < 0.001 |
| Cancer, % | 774 (30.9%) | 188 (29.7%) | 0.587 | 280 (16.7%) | 56 (18.9%) | 0.348 |
| COPD, % | 311 (12.4%) | 48 (7.6%) | 0.001 | 174 (7.3%) | 41 (7.8%) | 0.658 |
| Valve_disease, % | 778 (31.0%) | 250 (39.6%) | < 0.001 | 156 (6.5%) | 43 (8.2%) | 0.031 |
| PH_min | 7.32 ± 0.10 | 7.23 ± 0.16 | < 0.001 | 7.30 ± 0.13 | 7.19 ± 0.16 | < 0.001 |
| BE_min, mmol/L | -2.63 ± 5.25 | -8.09 ± 8.27 | < 0.001 | -3.39 ± 7.01 | -8.06 ± 8.47 | < 0.001 |
| BE_max, mmol/L | 0.98 ± 4.41 | -1.80 ± 6.05 | < 0.001 | 2.65 ± 5.94 | 0.90 ± 7.99 | < 0.001 |
| AG_min, mmol/L | 13.1 ± 3.2 | 15.9 ± 5.4 | < 0.001 | 9.5 ± 3.8 | 11.6 ± 5.3 | < 0.001 |
| AG_max, mmol/L | 16.5 ± 4.5 | 21.0 ± 7.2 | < 0.001 | 12.8 ± 5.1 | 17.3 ± 7.1 | < 0.001 |
| HR_min, bpm | 68.0 ± 14.4 | 70.0 ± 18.9 | 0.004 | 71.7 ± 20.2 | 74.2 ± 25.5 | 0.028 |
| HR_max, bpm | 100.7 ± 22.7 | 111.3 ± 27.1 | < 0.001 | 90.3 ± 24.2 | 98.3 ± 25.9 | < 0.001 |
| SBP_min, mmHg | 90.1 ± 15.7 | 77.7 ± 20.0 | < 0.001 | 112.2 ± 26.7 | 101.4 ± 30.9 | < 0.001 |
| MBP_min, mmHg | 58.3 ± 12.7 | 49.6 ± 16.5 | < 0.001 | 78.5 ± 18.9 | 70.2 ± 22.9 | < 0.001 |
| RR_min, bpm | 12.2 ± 3.5 | 13.5 ± 4.8 | < 0.001 | 18.1 ± 5.8 | 18.6 ± 8.3 | < 0.001 |
| RR_max, bpm | 27.7 ± 6.1 | 30.4 ± 7.6 | < 0.001 | 20.9 ± 6.3 | 23.1 ± 8.9 | < 0.001 |
| Temperature_min, ℃ | 36.3 ± 0.7 | 35.9 ± 1.1 | < 0.001 | 35.8 ± 0.9 | 34.9 ± 1.4 | < 0.001 |
| Temperature_max, ℃ | 37.2 ± 0.7 | 36.9 ± 1.1 | < 0.001 | 37.7 ± 1.2 | 38.0 ± 1.5 | < 0.001 |
| SpO_2__min | 91.9 ± 5.4 | 87.3 ± 13.1 | < 0.001 | 92.1 ± 9.3 | 85.1 ± 17.9 | < 0.001 |
| CVP_min, mmHg | 8.5 ± 3.2 | 9.9 ± 4.1 | < 0.001 | 9.7 ± 6.6 | 12.1 ± 7.3 | 0.003 |
| CVP_max, mmHg | 12.4 ± 3.4 | 13.5 ± 3.9 | < 0.001 | 14.6 ± 7.4 | 18.3 ± 7.7 | < 0.001 |
| Glucose_min, mg/dL | 110.3 ± 33.3 | 122.6 ± 51.4 | < 0.001 | 123.9 ± 45.6 | 135.8 ± 67.1 | < 0.001 |
| HGB_min, mg/dL | 10.4 ± 2.3 | 9.6 ± 2.3 | < 0.001 | 11.6 ± 2.5 | 10.6 ± 2.5 | < 0.001 |
| HGB_max, mg/dL | 11.6 ± 2.2 | 11.2 ± 2.2 | < 0.001 | 12.8 ± 2.4 | 12.1 ± 2.4 | < 0.001 |
| WBC_min, x 10^9/L | 9.9 ± 4.7 | 11.8 ± 6.6 | < 0.001 | 10.1 ± 6.1 | 12.0 ± 6.5 | < 0.001 |
| RBC_min, x 10^12/L | 2.87 ± 0.73 | 2.81 ± 1.75 | 0.199 | 3.59 ± 0.83 | 3.23 ± 0.84 | < 0.001 |
| RBC_max, x 10^12/L | 4.65 ± 0.68 | 4.35 ± 0.72 | < 0.001 | 4.31 ± 0.75 | 4.09 ± 0.79 | < 0.001 |
| BUN_min, mg/dL | 25.8 ± 20.3 | 36.7 ± 26.7 | < 0.001 | 22.7 ± 16.6 | 30.9 ± 22.8 | < 0.001 |
| APTT_min, s | 33.4 ± 13.7 | 38.9 ± 20.9 | < 0.001 | 33.9 ± 13.2 | 37.8 ± 14.8 | < 0.001 |
| PT_max, s | 17.8 ± 7.6 | 22.1 ± 10.1 | < 0.001 | 18.1 ± 10.4 | 22.9 ± 14.4 | < 0.001 |
| Phosphorus_min, mg/dL | 2.68 ± 0.85 | 2.97 ± 1.59 | < 0.001 | 2.84 ± 1.12 | 3.37 ± 2.02 | < 0.001 |
| Phosphorus_max, mg/dL | 4.40 ± 1.41 | 5.53 ± 2.45 | < 0.001 | 4.22 ± 1.60 | 5.65 ± 2.39 | < 0.001 |
| Chlorine_min, mmol/L | 101.1 ± 6.2 | 100.1 ± 7.5 | < 0.001 | 101.5 ± 5.8 | 99.7 ± 7.4 | < 0.001 |
| Inotropic, % | 999 (39.8%) | 477 (75.5%) | < 0.001 | 512 (21.3%) | 309 (58.9%) | < 0.001 |
| AAD, % | 2311 (92.1%) | 517 (81.8%) | < 0.001 | 1338 (55.8%) | 318 (60.6%) | 0.044 |

GCS: Glasgow coma scale; LOS_ICU: length of stay in intensive care unit; HF: heart failure; OMI: old myocardial infarction; SCA: sudden cardiac arrest; COPD: chronic obstructive pulmonary disease; PH: potential of hydrogen; BE: base excess; AG: anion gap; HR: heart rate; SBP: systolic blood pressure; MBP: mean blood pressure; RR: respiratory rate; CVP: central venous pressure; HGB: hemoglobin; WBC: white blood cell; RBC: red blood cell; BUN: blood urea nitrogen; APTT: activated partial thromboplastin time; PT: prothrombin time; AAD: antiarrhythmic drugs
