# Supplementary material for: Development and Validation of Machine Learning–Based Models to Predict In-Hospital Mortality in Life-Threatening Ventricular Arrhythmias: Retrospective Cohort Study
Source: J Med Internet Res. 2023 Nov 15;25:e47664. doi: 10.2196/47664 (PMC10687678; doi:10.2196/47664)
Supplement: Multimedia Appendix 2 [file jmir_v25i1e47664_app2.docx]

| **Multimedia Appendix 2**. All Variables Used in Machine Learning Models | | | |
| --- | --- | --- | --- |
| All included data  (N = 3140) | Percent Missing | Imputation Method | Description |
| **Demographics** | | | |
| Age | 0.0 | / |  |
| Male | 0.0 | / |  |
| Weight | 0.0 | Mean value |  |
| Height | 45.1 | Excluded |  |
| Race | 0.0 | / |  |
| GCS score | 0.0 | / | Glasgow Coma Scale |
| LOS | 0 | / | Length of stay in hospital prior to life-threatening ventricular arrhythmias |
| **Vital signs and invasive monitoring** | | | |
| Temperature* | 8.2 | Multiple imputation |  |
| HR* | 0.2 | Mean value | Heart rate |
| SBP* | 1.1 | Mean value | Systolic blood pressure |
| DBP* | 1.1 | Mean value | Diastolic blood pressure |
| MAP* | 0.3 | Mean value | Mean aortic pressure |
| RR* | 0.3 | Mean value | Respiratory rate |
| SpO2* | 0.6 | Mean value | Saturation of pulse oxygen |
| UO* | 3.3 | Mean value | Urine output |
| CVP* | 39.6 | Multiple imputation | Central venous pressure |
| CO | 80.2 | Excluded | Cardiac output |
| PCWP | 92.3 | Excluded | Pulmonary capillary wedge pressure |
| **Comorbidities** | | | |
| SCA | 0 | / | Sudden cardiac arrest |
| Hypertension | 0 | / |  |
| CHF | 0 | / | Congestive heart failure |
| AF | 0 | / | Atrial fibrillation |
| AMI | 0 | / | Acute myocardial infarction |
| OMI | 0 | / | Old myocardial infarction |
| NICM | 0 | / | Non-ischemic cardiomyopathy, including hypertrophic cardiomyopathy, restrained cardiomyopathy, non-ischemic diastolic cardiomyopathy |
| Angina pectoris | 0 | / |  |
| Valvular disease | 0 | / |  |
| AKI | 0 | / | Acute kidney injury |
| CKD | 0 | / | Chronic kidney disease |
| Hepatitis | 0 | / |  |
| Liver cirrhosis | 0 | / |  |
| Stroke | 0 | / | Including cerebral infarction and hemorrhage |
| COPD | 0 | / | Chronic obstructive pulmonary disease |
| Pneumonia | 0 | / |  |
| Cancer | 0 | / |  |
| Anemia | 0 | / |  |
| Diabetes | 0 | / |  |
| Syncope | 0 | / |  |
| Dyslipidemia | 0 | / |  |
| PE | 0 | / | Pulmonary embolism |
| **Laboratory tests** | | | |
| Lactate | 44.4 | Excluded |  |
| PH* | 38.0 | Multiple imputation |  |
| PaO2* | 38.0 | Multiple imputation |  |
| PaCO2* | 38.0 | Multiple imputation |  |
| AaDO2 | 54.1 | Excluded | Alveolar-arterial oxygen difference |
| PFR | 54.1 | Excluded | PaO2 FiO2 ratio |
| BE* | 38.0 | Multiple imputation | Base excess |
| Bicarbonate | 98.4 | Excluded |  |
| Total_CO2* | 38.0 | Multiple imputation |  |
| AG* | 0.6 | Mean value | Anion gap |
| WBC* | 0.7 | Mean value | White blood cell count |
| RBC* | 7.8 | Multiple imputation | Red blood cell count |
| PLT* | 0.8 | Mean value | Platelet count |
| Hemoglobin* | 0.7 | Mean value |  |
| Hematocrit* | 0.6 | Mean value |  |
| CRP | 72.4 | Excluded | C-reaction protein |
| D-dimer | 99.7 | Excluded |  |
| Fibrinogen | 71.6 | Excluded |  |
| Thrombin | 99.9 | Excluded |  |
| INR* | 6.3 | Multiple imputation | International standard ratio |
| APTT* | 7.0 | Multiple imputation | Activated partial thromboplastin time |
| Total protein | 97.8 | Excluded |  |
| Albumin | 59.4 | Excluded |  |
| Globulin | 98.7 | Excluded |  |
| SCr* | 0.3 | Mean value | Serum creatinine |
| BUN* | 0.3 | Mean value | blood urine nitrogen |
| Troponin T | 52.1 | Excluded |  |
| CK-MB | 73.4 | Excluded | Creatine kinase-muscle/brain |
| NT-pro BNP | 50.6 | Excluded | N-terminal-pro hormone B-type natriuretic peptide |
| ALT* | 39.3 | Multiple imputation | alanine transaminase |
| AST* | 39.0 | Multiple imputation | aspartate transaminase |
| Amylase | 92.3 | Excluded |  |
| TBIL | 40.8 | Excluded | Total bilirubin |
| DBIL | 95.4 | Excluded | Direct bilirubin |
| IBIL | 95.8 | Excluded | Indirect bilirubin |
| GGT | 99.5 | Excluded | Gamma-glutamyl transpeptidase |
| CK | 57.3 | Excluded | Creatine kinase |
| CK-MB | 52.6 | Excluded | Creatine kinase myocardial band |
| LDH | 62.8 | Excluded | Lactate dehydrogenase |
| Uric acid | 80.5 | Excluded |  |
| Glucose* | 1.5 | Mean value |  |
| Sodium* | 0.5 | Mean value |  |
| Potassium* | 0.5 | Mean value |  |
| Calcium* | 6.3 | Multiple imputation |  |
| Chlorine* | 0.5 | Mean value |  |
| Phosphorus* | 11.8 | Multiple imputation |  |
| Magnesium* | 8.9 | Multiple imputation |  |
| **Treatments** | | | |
| MV | 0 | / | Mechanical ventilation |
| RRT | 0 | / | Renal replacement therapy |
| CABG | 0 | / | Coronary artery bypass graft |
| PCI | 0 | / | Percutaneous coronary intervention |
| Inotropic | 0 | / | Including dopamine, dobutamine, epinephrine, norepinephrine, phenylephrine |
| Antibiotic | 0 | / |  |
| AAD | 0 | / | Anti-arrhythmia drug, including amiodarone, beta-blockers, Class Ib and Class Ic drugs. |

* The minimum and maximum values of these variables were included to build the prediction models.
