# Supplementary material for: Development and Validation of Machine Learning–Based Models to Predict In-Hospital Mortality in Life-Threatening Ventricular Arrhythmias: Retrospective Cohort Study
Source: J Med Internet Res. 2023 Nov 15;25:e47664. doi: 10.2196/47664 (PMC10687678; doi:10.2196/47664)
Supplement: Multimedia Appendix 3 [file jmir_v25i1e47664_app3.docx]

| **Multimedia Appendix 3.** Excluded variables after feature selection by LASSO | |
| --- | --- |
| Variables | Coefficient of LASSO penalty |
| HTN | 0 |
| DM | 0 |
| AF | 0 |
| AKI | 0 |
| AMI | 0 |
| CKD | 0 |
| Cl_max | 0 |
| Stroke | 0 |
| PH_max | 0 |
| PO2_min | 0 |
| PO2_max | 0 |
| PCO2_min | 0 |
| PCO2_max | 0 |
| Total_CO2_min | 0 |
| Total_CO2_max | 0 |
| SBP_max | 0 |
| DBP_min | 0 |
| DBP_max | 0 |
| MBP_max | 0 |
| SpO2_max | 0 |
| Glucose_max | 0 |
| Hematocrit_min | 0 |
| Hematocrit_max | 0 |
| Platelets_min | 0 |
| Platelets_max | 0 |
| WBC_max | 0 |
| BUN_max | 0 |
| Calcium_min | 0 |
| Calcium_max | 0 |
| Creatinine_min | 0 |
| Creatinine_max | 0 |
| Sodium_min | 0 |
| Sodium_max | 0 |
| Potassium_min | 0 |
| Potassium_max | 0 |
| INR_min | 0 |
| INR_max | 0 |
| APTT_max | 0 |
| PT_min | 0 |
| ALT_min | 0 |
| ALT_max | 0 |
| AST_min | 0 |
| AST_max | 0 |
| PE | 0 |
| STEMI | 0 |
| Syncope | 0 |
| Sepsis | 0 |
| Pneumonia | 0 |
| Dyslipidemia | 0 |
| Live_disease | 0 |
| Male | 0 |
| Antibiotics | 0 |
| Post_CABG | 0 |
| Post_PCI | 0 |

The abbreviations are as same in Multimedia Appendix 2
