# Supplementary material for: Development and Validation of Machine Learning–Based Models to Predict In-Hospital Mortality in Life-Threatening Ventricular Arrhythmias: Retrospective Cohort Study
Source: J Med Internet Res. 2023 Nov 15;25:e47664. doi: 10.2196/47664 (PMC10687678; doi:10.2196/47664)
Supplement: Multimedia Appendix 4 [file jmir_v25i1e47664_app4.docx]

**
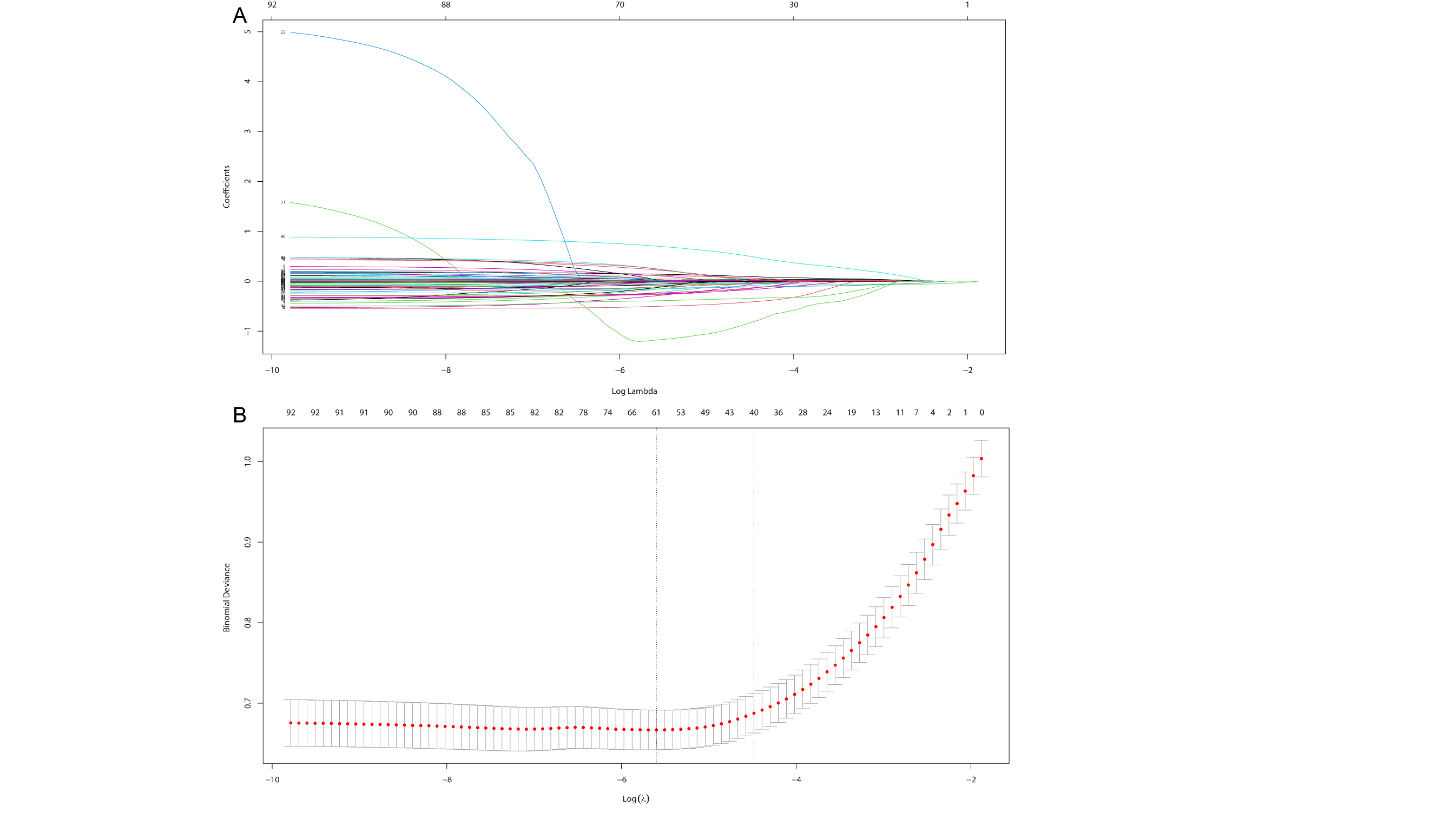
Multimedia Appendix 4**. Feature selection by LASSO analysis. (**A**) Plots for LASSO analysis coefficients; (B) Cross validation plot for the penalty term.
