# Supplementary material for: Development and Validation of Machine Learning–Based Models to Predict In-Hospital Mortality in Life-Threatening Ventricular Arrhythmias: Retrospective Cohort Study
Source: J Med Internet Res. 2023 Nov 15;25:e47664. doi: 10.2196/47664 (PMC10687678; doi:10.2196/47664)
Supplement: Multimedia Appendix 5 [file jmir_v25i1e47664_app5.docx]

**Multimedia Appendix 5**. Characteristics of the included machine learning algorithms.

| Model | Type | Algorithm Classification | Advantages | Disadvantages |
| --- | --- | --- | --- | --- |
| CatBoost | Supervised ML | Ensemble learning  (Boosting) | 1. Excellent prediction performance  2. Less overfitting, good robustness  3. Handling missing values automatically | 1. Long calculating time  2. Requiring large RAM |
| LightGBM | Supervised ML | Ensemble learning (Boosting) | 1. High learning efficiency  2. Parallel learning  3. Could handle high-dimensional data | 1. Abnormal values will highly affect the prediction performance |
| Random Forest | Supervised ML | Ensemble learning (Bagging) | 1. Could predict classification and regression outcomes  2. Less overfitting, good robustness  3. Could handle high-dimensional data | 1. Long calculating time  2. Requiring large RAM |
| Logistic Regression | Supervised ML | Interpretive algorithm | 1. High learning efficiency  2. Simple algorithm, fast convergence  3. Good interpretability | 1. Limited prediction performance  2. Unable to handle nonlinear issue  3. Unable to handle unbalance data |
| BP Neural Network | Supervised ML | Neural network | 1. Excellent nonlinear mapping capability  2. High self-learning and self-adaption ability  3. Good generalization ability | 1. Local minimum problem  2. Limited learning efficiency |

ML: machine learning; RAM: random access memory; BP: back propagation.
