# Supplementary material for: Development and Validation of Machine Learning–Based Models to Predict In-Hospital Mortality in Life-Threatening Ventricular Arrhythmias: Retrospective Cohort Study
Source: J Med Internet Res. 2023 Nov 15;25:e47664. doi: 10.2196/47664 (PMC10687678; doi:10.2196/47664)
Supplement: Multimedia Appendix 6 [file jmir_v25i1e47664_app6.docx]

| **Multimedia Appendix 6.** Comparisons of AUCs of different models by Delong Test | | |
| --- | --- | --- |
|  | Z score | P value |
| CatBoost vs. LODS | 7.246 | < 0.001 |
| CatBoost vs. SAPS-II | 6.063 | < 0.001 |
| LightGBM vs. LODS | 6.636 | < 0.001 |
| LightGBM vs. SAPS-II | 5.358 | < 0.001 |
| RF vs. LODS | 5.349 | < 0.001 |
| RF vs. SAPS-II | 4.251 | < 0.001 |
| LR vs. LODS | 6.189 | < 0.001 |
| LR vs. SAPS-II | 5.262 | < 0.001 |
| BP-NN vs. LODS | 6.741 | < 0.001 |
| BP-NN vs. SAPS-II | 6.128 | < 0.001 |
| LODS vs. SAPS-II | -0.836 | 0.403 |
