# Supplementary figures and images for: Development and Validation of Machine Learning–Based Models to Predict In-Hospital Mortality in Life-Threatening Ventricular Arrhythmias: Retrospective Cohort Study
Source: J Med Internet Res. 2023 Nov 15;25:e47664. doi: 10.2196/47664 (PMC10687678; doi:10.2196/47664)

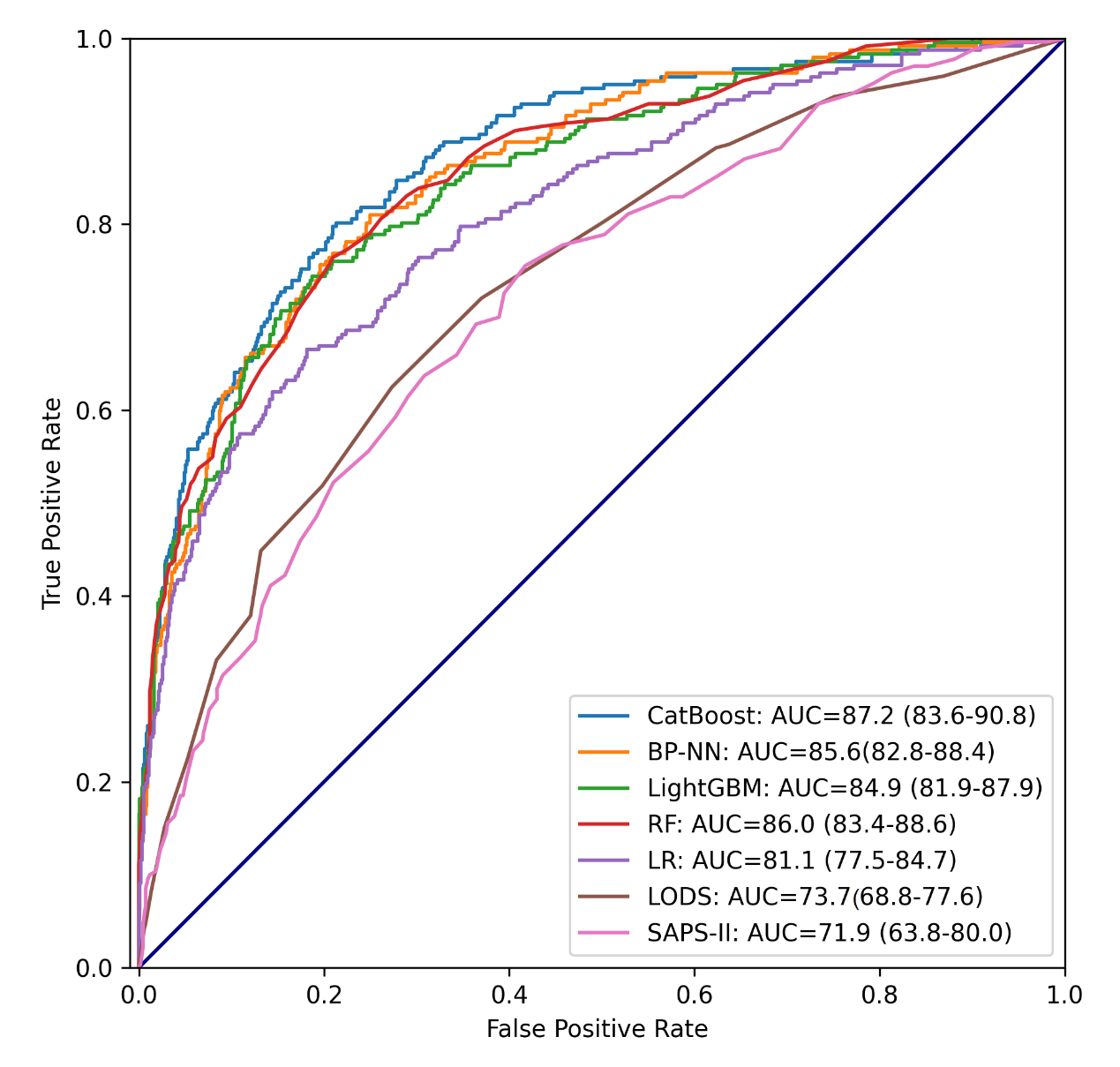


**Multimedia Appendix 7.** External validation of different models.

Supplement: Multimedia Appendix 7 [file jmir_v25i1e47664_app7.docx]
